# Advancing Mental Health Care: A Comprehensive Review of Digital Tools and Technologies for Enhancing Diagnosis, Treatment, and Wellness

**DOI:** 10.1002/hcs2.70018

**Published:** 2025-05-31

**Authors:** Muhammad Khalid Anser, Agha Amad Nabi, Ishfaq Ahmad, Muhammad Moinuddin Qazi Abro, Khalid Zaman

**Affiliations:** ^1^ Department of Economics, Faculty of Economics and Administrative Sciences Recep Tayyip Erdoğan University Rize Turkey; ^2^ Department of Business Administration Government College University Hyderabad Hyderabad, Sindh Pakistan; ^3^ Lahore Business School (LBS), Faculty of Management Sciences The University of Lahore Lahore Pakistan; ^4^ Department of Production and Systems Engineering Universidade Tecnológica Federal do Paraná (UTFPR) Pato Branco Campus Brazil; ^5^ Department of Economics The University of Haripur Haripur Khyber Pakhtunkhwa Pakistan

**Keywords:** artificial intelligence, digital mental health tools, mental health interventions, online therapy platforms, virtual reality therapy, wearable devices

## Abstract

An individual's mental health influences their capacity to think effectively, feel emotionally stable, and perform daily activities. As mental health concerns become more prevalent worldwide, new awareness and diagnostic and treatment tactics are needed. Digital tools and technology are helping solve these problems by providing scalable, tailored solutions for large populations. This detailed review examines mental health‐promoting internet tools. Smartphone applications, web‐based therapy systems, wearable tech, artificial intelligence‐powered resources, and virtual reality (VR) technologies were evaluated for efficacy and side effects. PubMed, PsycINFO, Scopus, IEEE Xplore, and Google Scholar were carefully searched. Search terms included “digital mental health tools,” “online therapy,” and “AI in mental health.” Randomized controlled trials, cohort studies, cross‐sectional studies, systematic reviews, and meta‐analyses of digital technology and mental health were included from among the literature published after 2010. Cognitive behavioral therapy methods, mood monitoring, and mindfulness exercises are among the numerous features of smartphone applications that have been demonstrated to mitigate symptoms of anxiety, depression, and tension. Online therapy platforms let marginalized individuals obtain therapy remotely. Wearable technology may detect heart rate, blood pressure, and sleep length, which may reveal mental health difficulties. Chatbots employ machine learning algorithms and natural language processing to deliver customized support and show promise for quick intervention. Exposure therapy for anxiety and trauma is increasingly using virtual reality environments. Although digital mental health therapies face challenges in relation to data privacy, limited long‐term efficacy, and technological inequality, digital technologies are modernizing mental healthcare. By offering inexpensive and effective alternatives to traditional therapies, digital technologies may help healthcare systems meet the growing demand for mental health services and overall well‐being.

## Graphical abstract

Digital tools support mental health through apps, online platforms, and wearables. This study aims to evaluate digital mental health tools' effectiveness. A systematic review was conducted across diverse databases. Apps like Headspace and Calm effectively manage stress and anxiety. AI tools like Woebot and Wysa offer personalized mental health support.

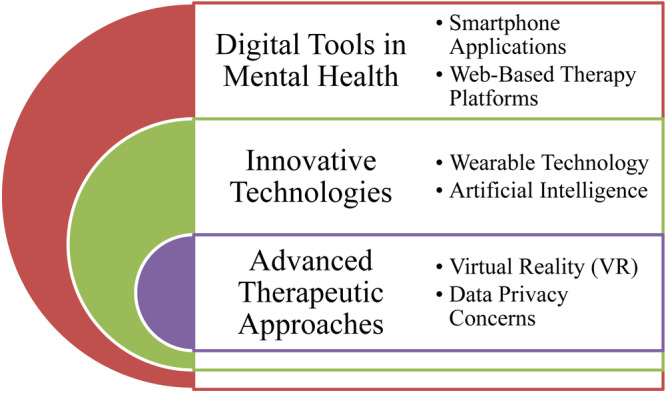

**Abbreviations**
CBT: cognitive behavioral therapy
NLP: natural language processing
PTSD: posttraumatic stress disorder
VR: virtual reality

## 1 Introduction

Tech‐driven “digital mental health tools” aid mental health screening, intervention, and monitoring via online media [1]. Advances in artificial intelligence (AI), smartphone applications, wearable tech, virtual reality (VR), and online therapy platforms make mental health treatment more accessible and practical [2]. A systematic approach to organize mental health web resources, including mobile health apps, includes self‐guided therapeutic modules, mindfulness exercises, mood monitoring, and cognitive behavioral therapy (CBT)‐based programs for mental health [3]. Patients may consult trained therapists online via video chats, short‐message service messages, and AI‐powered psychotherapy. AI‐powered chatbots and diagnostic aides employ natural language processing (NLP) and machine learning (ML) to deliver real‐time mental health help, symptom assessment, and therapy ideas [4]. Biosensors and wearable tech—Fitbits and Apple Watches—measure heart rate variability, sleep patterns, and stress levels, which may assist in diagnosing mental health disorders [5]. Augmented reality (AR) and VR treatments include immersive digital environments for posttraumatic stress disorder (PTSD), relaxation, and exposure therapy [6].

Healthy minds allow individuals to think, feel, and act in ways that benefit their health. Thoughts, emotions, and social interactions affect stress management, relationship formation, and decision‐making [7]. The World Health Organization [8] reports a rise in mental health disorders affecting individuals of all ages, backgrounds, and places. Mental health difficulties, including depression, anxiety, bipolar illness, and schizophrenia, affect people's well‐being, productivity, and everyday life. The rising incidence of mental health concerns requires innovative preventive, treatment, and promotion measures. Traditionally, mental health treatment has included medication, in‐person therapy, and institutionalization. These techniques are effective but have limitations, including high costs, societal stigma, accessibility issues, and a lack of mental health professionals [9, 10]. These issues were serious before the coronavirus disease 2019 pandemic, and today, complementary and alternative mental health treatment is needed more than ever [11]. Digital tools and technology are powerful mental health allies because of their scalability, accessibility, and low cost [12]. Apps, wearable devices, online support groups, teletherapy platforms, and AI‐powered treatments are all part of the digital mental health toolset. These tools leverage modern technology to improve mental health management and monitoring [13]. Stress and anxiety treatment apps like Headspace and Calm provide guided meditations and mindfulness exercises [14]. With BetterHelp and Talkspace, users may connect with licensed therapists via video calls, chat, or phone [15]. Wearable electronics like Apple Watch and Fitbit detect physiological data like heart rate and sleep patterns, which may indicate mental wellness [16].

Digital technology in mental health therapy has several advantages. It removes temporal and geographical barriers to providing mental health treatment to those who need it, which is particularly useful in rural areas with few mental health services. Digital technology may make mental healthcare cheaper and more scalable, thereby revolutionizing it. It also enables users to customize and adapt features to their needs, improving engagement and effectiveness [17]. However, digital mental health tools face barriers to broad adoption. Technology access remains unequal owing to differences in age, region, and socioeconomic status, known as the digital divide. Mental health information is sensitive; thus, privacy and security are crucial. Data security involves strict precautions to prevent unauthorized access [18]. Even though many digital technologies show promise, further research is needed to evaluate their efficacy and long‐term benefits. Despite these barriers, online options for mental health improvement remain promising. This study reviews digital mental health tools, weighs their merits and downsides, and proposes improvements. This paper reviews digital treatments and suggests methods to enhance them, contributing to the ongoing discussion on improving mental healthcare using technology.

Digital technologies have changed mental healthcare in the past few years, delivering accessible and scalable diagnosis, treatment, and support solutions. Smartphone applications for CBT and AI‐powered chatbots have been studied as psychological support systems [19, 20]. Studies suggest that digital mental health solutions may reduce stress, unhappiness, and anxiety. However, although the literature is increasing, several important gaps remain. First, although research has studied individual treatments, few comparative studies of the effectiveness and adverse effects of digital therapies exist [21, 22]. Most studies overlook digital mental health, focusing instead on technology interactions [23, 24], making it hard to determine which instruments function best for different populations and mental health conditions. To address this gap, this study evaluates smartphone applications, online therapy platforms, AI‐driven tools, and VR treatments, assessing their merits and downsides. Second, previous research demonstrates high attrition rates for mobile‐based mental health therapy [25], raising concerns about their long‐term viability. The study synthesizes long‐term engagement analyses, identifying factors that help consumers persist with digital mental health treatments. Third, digital literacy and access disparities persist even as digital technologies have expanded mental health help. Some studies show that low‐income older adults, who are already at a disadvantage, may have more trouble using digital mental health services [26, 27]. Data privacy, ethics, and regulation are inadequately addressed in contemporary research. This study examines these concerns, which may inform policy and solutions to improve access and privacy.

Because of global mental health challenges, digital resources in mental health therapy have increased. These treatments' accessibility, long‐term engagement, and comparative efficacy have yet to be assessed. This study evaluates smartphone applications, online therapy platforms, AI‐powered tools, wearable gadgets, and VR therapies to fill these gaps. It is important to examine three things: first, the efficacy of these digital tools in alleviating stress, anxiety, and depression; second, potential pitfalls and solutions; and third, the factors that influence user engagement, accessibility, and data privacy issues. This study's exhaustive digital mental health therapy overview draws on various analyses. The results illuminate these strategies' potential, scalability, and influence on mental healthcare. This study contributes to discussions of digital health transformation involving many technological strategies [28]. It contributes to the debate over harnessing digital innovation to enhance mental health services and decrease healthcare access disparities.

## 2 Expanding the Scope of Digital Mental Health Tools

Digital mental health tools are increasing and span a broad spectrum of technology that supports cognitive health. Mobile apps are popular digital mental health resources, offering mood tracking, meditation, CBT, and psychoeducation. Moodfit and Daylio help track moods, identify patterns, and develop coping skills [29]. Smiling Mind and Insight Timer offer guided meditations and mindfulness exercises to reduce stress and improve emotional control. Teletherapy platforms are another significant online mental health resource [30]. Users can schedule phone, chat, or video therapy sessions with qualified mental health professionals on these sites. Teletherapy was effective during the coronavirus disease 2019 pandemic, allowing patients to receive therapy without being near others. Amwell and MDLIVE offer broad teletherapy services for trauma, anxiety, and depression. Wearable gear also aids digital mental health tools [31]. Smart watches and fitness trackers track heart rate, sleep, and activity. Real‐time data from wearables can illuminate the relationship between habits and mental health. The Garmin Vivosmart tracks stress by measuring heart rate variability, whereas the Oura Ring assesses sleep quality and well‐being [32, 33]. Several online support groups and forums allow people to share their experiences and offer encouragement. Community members of HealthUnlocked can share stories and seek advice and support from people with mental health challenges. These groups can reduce stigma and provide a place to belong for such patients. Digital mental health tools also benefit from AI and ML advances. AI‐powered chatbots and virtual therapists like Woebot and Wysa use NLP and ML algorithms to provide rapid help and advice [34]. These AI systems can help detect and treat mental health issues by talking to people, providing CBT, and measuring mood changes.

The literature on digital mental health therapies has advanced to include smartphone applications, AI‐powered chatbots, wearable health monitors, and VR therapy. Studies show that these technologies may help manage mental health by supporting cognitive behavioral treatment [35], real‐time crisis assistance [36], and remote physiological monitoring of mental health markers [37]. However, even with these advancements, huge gaps remain. Most existing studies focus on single digital therapies rather than comparing their effectiveness or place within mental healthcare paradigms. These technologies have potential, but the literature does not often address user engagement and retention, which are crucial to long‐term mental health benefits. Inadequate research on digital mental health treatment equity and accessibility is another issue. Studies suggest that data privacy concerns, limited technical competence, and socioeconomic inequalities limit digital technology's spread [38, 39]. The morality of AI‐powered mental health therapies, notably in terms of trustworthiness, accountability, and algorithmic bias, is also disputed [40]. These challenges underscore the need to study ways to enhance digital mental health treatments for diverse populations while ensuring fair and ethical deployment. This study carefully evaluates digital mental health therapy's effectiveness, risks, and accessibility to address these gaps. Our research aims to evaluate the efficacy and user engagement of various digital interventions, examine the privacy and ethical issues raised by data‐intensive and AI‐driven tools, and propose solutions to underserved populations' lack of access to digital mental health services. The research contributes to discussions on digital health transformation and offers a framework for assessing and enhancing digital therapies.

## 3 The Benefits of Digital Tools for Mental Health Promotion

Digital tools offer innovative solutions to persistent challenges in mental healthcare, in turn enhancing mental health in various ways. A primary advantage is increased accessibility. Digital tools have “leveled the playing field” for mental health support services by removing time, location, and accessibility barriers, which is crucial because disadvantaged and rural populations have limited mental health interventions [41]. Teletherapy platforms allow rural residents to receive care without traveling far. Affordable digital mental health tools are another advantage. Facets of mental healthcare and therapy can be costly, including medicine, travel, and the therapy itself. Digital instruments are often cheaper or more accessible [42]. Mobile apps often have a free version with limited functionality and a premium version with more features. Because of its affordability, mental health treatment is available to those who cannot afford conventional therapy. Digital technologies provide enormous scope for personalization and flexibility. Many digital mental health solutions are user‐friendly and customizable. Meditation applications offer guided meditations to promote sleep and reduce anxiety [43]. Mood‐tracking applications with tailored reminders and goals can help users maintain their mental health. Individualized tools are more likely to be used and maximized. Digital technologies provide anonymity and privacy, making it easier for people to seek aid. Because of stigma, patients with mental health challenges are less inclined to seek in‐person therapy [44]. Digital tools allow people to seek aid anonymously without fear of judgment. Users can use anonymous online support groups or AI‐powered chatbots to discuss sensitive topics and obtain help.

Employing digital mental health solutions has pros and cons. One major issue is the digital divide, i.e., the gap in digital technology access between the rich and the poor. Age, education, location, financial class, and geography affect digital technology access. Internet connectivity, digital literacy, and smartphone access may prevent low‐income households, rural areas, and developing countries from accessing digital mental health resources [45]. Digital mental health tools must prioritize privacy and security. People may avoid digital technologies because of concerns about data breaches or exploitation, especially regarding sensitive mental health data. Implementing strict privacy regulations, encrypted data storage, and other security measures is essential to protect user data and build credibility. Additionally, developers must comply with all laws and regulations, including the EU's General Data Protection Regulation and the USA's Health Insurance Portability and Accountability Act [46]. Digital mental health goods must also be evaluated for efficacy and evidence. Despite their potential, digital technologies need further research and clinical trials to determine their usefulness and long‐term benefits. Because new tools and updates are released constantly, keeping up with research and validation can take time and effort. The variability in digital instruments' features and functions complicates efficacy evaluation. Digital mental health solutions must be evaluated using defined frameworks and standards to be effective and evidence‐based [47]. Finally, digital tools and technology can make mental healthcare more accessible, inexpensive, and personalized. Wearable technologies, online support groups, teletherapy, mobile apps, and AI‐driven initiatives could improve mental healthcare and reach marginalized people. Digital mental health aids must overcome the digital gap, privacy and security concerns, and a lack of research to realize their full potential. New technology must be integrated into mental health treatment to meet rising demand and improve well‐being [48]. This review paper emphasizes the importance of employing digital tools for mental healthcare. It urges further research, new policies, and novel practices to ensure their availability, efficacy, and safety.

## 4 Case Studies and Examples

This study encompasses three case studies, namely Headspace, BetterHelp, and Fitbit because they have mental health applications and are popular and effective. Many clinical investigations have proven that Headspace's mindfulness‐based treatments reduce anxiety and stress. BetterHelp's ease of use and scalability make it the best online mental health therapy. Fitbit, a popular wearable tech device, was included because its ability to track physiological indicators like heart rate and sleep patterns is pertinent to mental health monitoring. Several items were used to evaluate digital mental health technologies' impact on mental health therapy.

### 4.1 Headspace

The famous meditation program Headspace offers guided meditations and mindfulness exercises to reduce stress and anxiety and improve mental health. Since its introduction in 2010, Headspace has had millions of users worldwide, making it popular mental health software. The app offers meditations for stress, focus, sleep, and anxiety [49]. Headspace provides guided meditations, ranging from a few minutes to many hours, for all experience levels. The software offers mindfulness exercises, such as mindful walking, breathing exercises, and body scans, which users can incorporate into their daily lives. It offers peaceful sounds, meditations, and sleep casts to assist users in falling asleep [50]. The program also offers recommendations on the basis of user interests, goals, and prior interactions. Headspace's easy‐to‐use design, engaging content, and mental health benefits have made it successful. A 10‐day Headspace course reduced stress and annoyance [51]. Other research found that 8 weeks of app usage reduced anxiety and increased positivity [52]. These findings demonstrate the app's potential for practical and accessible mental health assistance.

### 4.2 BetterHelp

BetterHelp, an online treatment site, matches distant patients with mental health challenges with trained therapists. BetterHelp swiftly became the top‐ranked online counseling service after launching its video chat, phone, and text treatment platform in 2013. The website helps patients with mental health challenges discover treatment by making it easier, cheaper, and more accessible [16]. Patients may communicate with therapists via video chat, phone, or encrypted texting. With subscription‐based pricing, treatment is cheaper than in‐person sessions. BetterHelp also ensures users' privacy and anonymity so they may be more likely to seek treatment from this site. BetterHelp's versatility, user‐friendliness, and high‐quality treatment services have contributed to its success [53]. According to one study, internet therapy is equally effective as in‐person treatment for certain mental health conditions [54]. A behavioral and cognitive psychotherapy study found that online CBT decreased depression and anxiety symptoms [55]. Another study found that online mental health therapy works. Because of its accessible and effective treatment, BetterHelp may benefit patients with mental health challenges [56].

### 4.3 Fitbit

Fitbit, a popular device, tracks heart rate, sleep, and exercise, revealing linkages thereof to mental wellness. Fitbit has been a leading fitness and health brand since 2007, with millions of users worldwide. In addition to measuring physical health, the device provides mental health data [57]. Fitbit's activity monitoring features count steps, calories, and active minutes, and track distances, to promote health. The device also tracks sleeping patterns to assess sleep quality and provides tips for improving it. Fitbit users may track their heartrate daily to assess stress, exercise intensity, and resting heartrate. Some Fitbit devices include guided breathing exercises and heartrate variability‐based stress monitoring [58]. Fitbit has successfully increased physical exercise and well‐being, which are linked to mental health. One study found that frequent exercisers had lower anxiety and depression rates [59]. Another found that better sleep improves mental health. Fitbit may help us monitor and analyze lifestyle changes to improve mental health [60].

Many digital tools, including Headspace, BetterHelp, and Fitbit, may assist with mental health. Because they are accessible, handy, and simple, these tools may help manage stress, anxiety, depression, and other mental health disorders. Technology provides innovative mental health treatment by removing stigma, cost, and accessibility barriers. Digital mental health technology may transform mental health treatment and boost global well‐being.

## 5 Methods

This systematic review examines mental health‐promoting internet tools through search, selection, and data extraction and synthesis steps, ensuring extensive and rigorous evaluation of internet mental health resources and literature.

### 5.1 Search Strategy

Many electronic databases were searched for digital mental health tool research and publications. The databases searched were as follows: (1) PubMed, which contains health‐related research publications and biological literature. (2) The psychological literature database PsycINFO, covering psychology and associated topics. (3) Scopus, a peer‐reviewed database of papers on numerous subjects. (4) IEEE Xplore, for healthcare technology and usage publications. (5) Google Scholar, which collates scholarly articles from many areas.

The search used keywords and phrases to collect all relevant research. The top searches included “digital mental health tools,” “mental health technologies,” “online therapy,” “mobile mental health apps,” “artificial intelligence in mental health,” “virtual reality therapy,” and “wearable technology and mental health.” We used Boolean operators to combine and exclude words, covering several digital mental health issues using keywords like “digital mental health tools AND mobile apps” and “AI in mental health OR virtual reality therapy.” A rigorous search of PubMed, PsycINFO, Scopus, IEEE Xplore, and Google Scholar allowed a comprehensive literature review. Digital mental health research publications were searched using keywords, Boolean operators, and various expressions. Table 1 shows the entire search strategy, including the key search words, Boolean combinations, and variations.

**Table 1:** Comprehensive search strategy for digital mental health tools.

Category	Primary keywords	Boolean operators & combinations
General terms	Digital mental health tools	“digital mental health tools AND technologies”
Therapeutic approaches	Online therapy, virtual therapy	“online therapy OR virtual therapy”
Mobile & app‐based	Mobile mental health apps, mental health apps	“mobile mental health apps AND CBT”
AI in mental health	Artificial intelligence in mental health	“AI in mental health OR machine learning for mental health”
Virtual reality therapy	Virtual reality therapy, VR‐based therapy	“virtual reality therapy AND anxiety treatment”
Wearable tech & tracking	Wearable technology and mental health	“wearable technology AND mental health tracking”
Mental health monitoring	Mental health tracking devices	“mental health tracking devices OR biometric mental health”
Machine learning & diagnosis	Machine learning in mental health diagnostics	“machine learning AND mental health diagnostics”

Abbreviation: CBT, cognitive behavioral therapy.

### 5.2 Selection Criteria

Research articles and studies had to be high quality and topical to pass the screening procedure. The inclusion and exclusion criteria follow.

Inclusion Criteria: (1) Research must be published after 2010 to incorporate the latest digital mental health technology advancements. (2) English must be used for consistency and clarity. (3) Permissible research types include randomized controlled trials (RCTs), cohort studies, cross‐sectional studies, meta‐analyses, and systematic reviews. (4) Mobile apps, online treatment platforms, wearable devices, AI, and VR research that develops, tests, and evaluates mental health‐promoting digital solutions are pertinent. (5) Research covering adults, teens, veterans, or individuals with long‐term mental health difficulties.

A publication date after 2010 was among the primary inclusion criteria. The rapid growth of digital mental health tools in the previous decade has proved crucial. According to many studies and meta‐analyses, smartphone applications, AI‐driven mental health assistance, VR therapy, and wearable technologies grew rapidly in the early 2010s [61, 62, 63]. Traditional teletherapy, used for cognitive development, did not use AI, ML, or real‐time mental health monitoring, the hallmarks of modern digital therapies. After 2010, consumer acceptance of mobile mental health solutions increased along with cloud‐based healthcare solutions and mobile app marketplaces [64]. This cut‐off date ensured that we analyzed only relevant and current digital interventions.

This study reviewed Science Citation Index (SCI) and Social Sciences Citation Index (SSCI) literature to ensure reliability and quality. SCI and SSCI publications undergo rigorous peer reviews and make important scientific contributions. We prioritized indexed sources to obtain studies with credible data, well‐validated methodologies, and an academic understanding of digital mental health. We also investigated systematic reviews, meta‐analyses, RCTs, and empirical research in high‐impact factor journals to ensure the scientific rigor and reliability of evaluations of the effectiveness of digital mental health tools.

Exclusion criteria: (1) Outdated data (published after 2010). (2) Articles not in English (because of translation issues). (3) Non‐peer‐reviewed articles like opinion pieces and case reports. (4) Research focusing on physical health only or ignoring mental health digital technology.

### 5.3 Data Extraction and Synthesis

Data were extracted from the studies systematically. Standardized data extraction forms guaranteed consistency and thoroughness. For each study, the following information was obtained: (1) Research details, including authors, year, and country. (2) Research methods, including survey size, demographics, and design (cohort vs. RCT). (3) Digital tool details, including technology type (AI, VR), purpose (therapy, monitoring, teaching), and form (e.g., mobile app, internet platform, wearable device). (4) The digital tool's effectiveness, user interaction, and impact on psychological well‐being. (5) The study's biases and limitations.A narrative synthesis was then performed on the extracted data to highlight research trends, similarities, and notable findings. This synthesis aimed to summarize digital mental health tools' current status, benefits, and future prospects. The following steps ensured synthesis validity and reliability: (6) The Newcastle–Ottawa Scale (for observational studies) and the Cochrane Risk of Bias Tool (for RCTs) were used to assess the publications. (7) Each study's themes and patterns were thematically analyzed to produce a coherent narrative.

**Figure 1:**
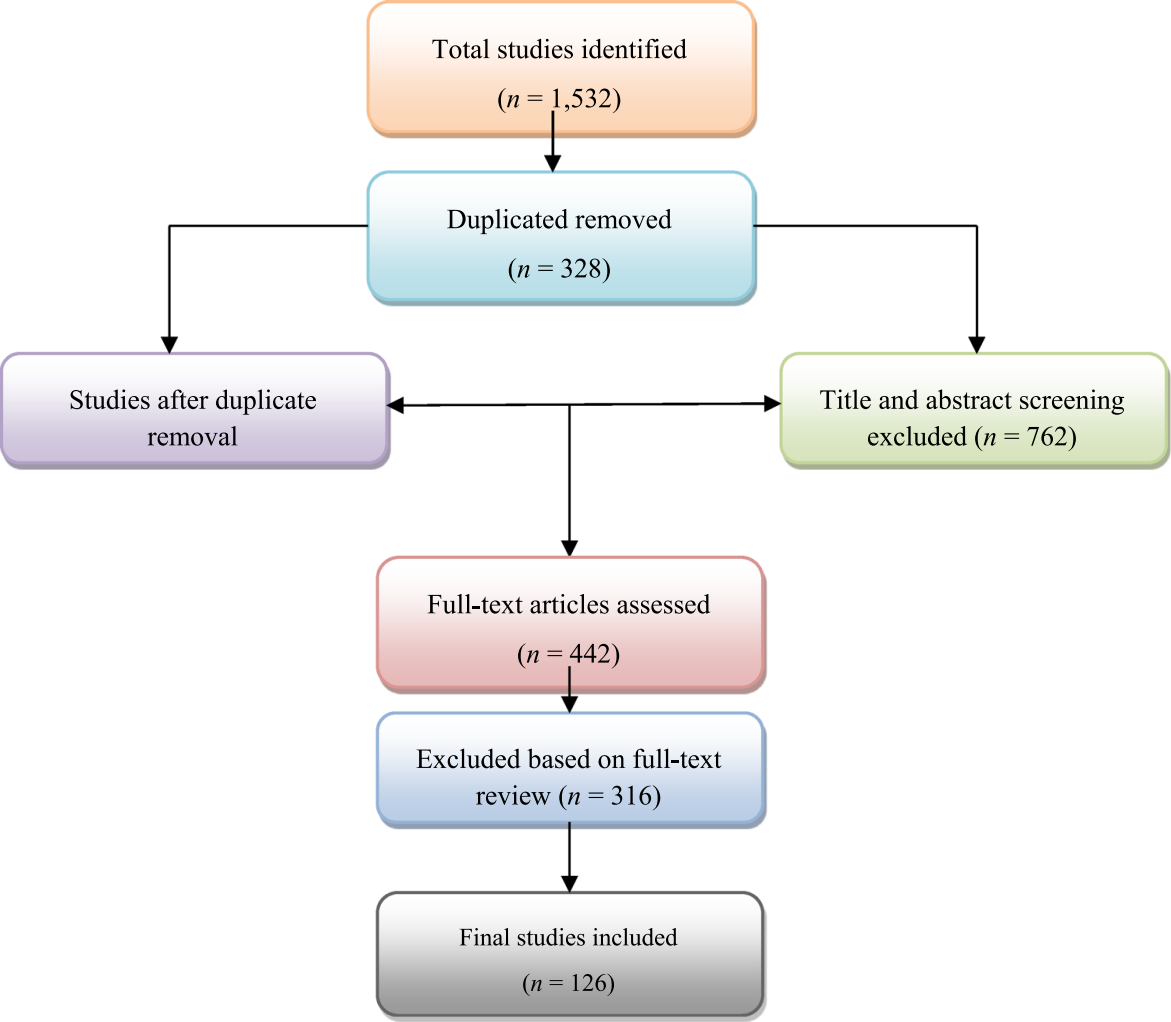
Preferred reporting items for systematic reviews and meta‐analyses flow diagram illustrating the study selection procedure.

A systematic search of PubMed, PsycINFO, Scopus, IEEE Xplore, and Google Scholar using the aforementioned search criteria yielded 1532 scholarly research papers. We employed Preferred Reporting Items for Systematic reviews and Meta‐Analyses‐compliant multistage screening to ensure relevant and well‐designed research was included. In the first stage, we identified and removed duplicate data, leaving 1204 trials for initial screening. Titles and abstracts were examined again using inclusion criteria including relevance to digital mental health, empirical research methods, and publication in SCI‐ or SSCI‐indexed journals. After excluding 762 publications because of a lack of relevance, methodological information, or peer review, 442 were left for full‐text examination. The third and last phase was complete full‐text paper examination. We eliminated research that did not follow clear methodological rules, had insufficient empirical data, or did not support this study's goals. Studies that discussed their claims theoretically without empirical evidence were also excluded. This comprehensive screening yielded 126 high‐quality publications for the final analysis. Because it included RCTs, cohort studies, systematic reviews, meta‐analyses, and large‐scale cross‐sectional studies, this study was based on strong empirical evidence and academic consensus. The study selection procedure is illustrated in the Preferred Reporting Items for Systematic reviews and Meta‐Analyses flow diagram (Figure 1).

This study's rigorous methodical approach allowed scientific review of the pros and cons of digital mental health therapy. The inclusion of high‐quality, peer‐reviewed publications from credible sources strengthens our findings.

This study included systematic reviews and meta‐analyses of digital mental health therapies. The studies reviewed past research to ensure they followed academic guidelines and contributed to the digital mental health technology literature. Mudiyanselage et al. [65] evaluated digital mental health applications' clinical practicality, effectiveness, and limitations. Their study examined the data supporting mobile health therapies, highlighting how these technologies may help meet the demand for mental healthcare while also revealing data privacy, regulatory monitoring, and user engagement issues. They used evidence‐based digital health treatment guidelines to evaluate mental health applications. Buragohain et al. [66] reviewed research on technology‐assisted mental health interventions, focusing on teletherapy platforms, AI‐powered mental health diagnostics, and CBT‐based apps. Although few long‐term follow‐up studies of digital therapies exist, their findings emphasize the need for longitudinal monitoring. This study critically evaluates digital mental health solutions' durability and long‐term consequences, offering a complete picture of their efficacy beyond initial user interaction. By following standards, the credibility and transparency of this study's methodology is increased. The theoretical and empirical validity of the study with respect to the academic debate on evidence‐based mental health technologies is ensured by mentioning systematic reviews and meta‐analyses [67, 68]. We included high‐impact studies to assess gaps, improvements, and possibilities in digital mental health therapy [69, 70]. This study's results contribute to digital mental healthcare theory and practice because of their methodological rigor and alignment with prior research.

## 6 Results

The results section details our findings on digital resources and mental health. The results are thematically categorized and summarized. Primary topics include new patterns, trends, and gaps, with a summary of the primary research findings provided in Table 2.

**Table 2:** Results of a systematic review of studies on digital tools for mental health promotion.

Authors	Study design	Digital tool description	Outcomes
Tan et al. [71]	Randomized controlled trial	Mobile app, AI‐driven	Reduced anxiety symptoms
Zeng et al. [72]	Cohort study	Online therapy platform	Improved depression outcomes
Zafar et al. [73]	Cross‐sectional study	Wearable device, AI‐driven	Enhanced mental health monitoring
Aggarwal et al. [74]	Systematic review	AI‐driven chatbots	Personalized mental health support
Kaur et al. [75]	Meta‐analysis	Virtual reality therapy	Reduced PTSD symptoms
Ulrich et al. [76]	Randomized controlled trial	Mobile app	Decreased stress levels
Stephenson et al. [77]	Cross‐sectional study	Online therapy platform	Effective remote therapy access
Blackman et al. [78]	Cohort study	Wearable device	Improved sleep patterns
Dvijotham et al. [79]	Randomized controlled trial	AI applications	Enhanced diagnostic accuracy
Freeman et al. [80]	Cohort study	Virtual reality therapy	Reduced phobia symptoms
Jeong et al. [81]	Randomized controlled trial	Mobile app	Alleviated depression symptoms
Oliveira et al. [82]	Systematic review	Online therapy platform	Reduced anxiety symptoms
Wakefield et al. [83]	Cross‐sectional study	Wearable device	Enhanced data collection
Barreveld et al. [84]	Randomized controlled trial	AI‐driven tools	Improved therapy outcomes
Schröder et al. [85]	Meta‐analysis	Virtual reality therapy	Decreased anxiety symptoms
Diano et al. [86]	Cross‐sectional study	Mobile app	Improved mood regulation
Ehlers et al. [87]	Cohort study	Online therapy platform	Effective PTSD treatment
Heizmann et al. [88]	Randomized controlled trial	Wearable device	Enhanced activity monitoring
Cabral et al. [89]	Cross‐sectional study	AI applications	Optimized treatment strategies
Hudon et al. [90]	Cohort study	Virtual reality therapy	Improved schizophrenia symptoms
Hilt et al. [91]	Randomized controlled trial	Mobile apps	Reduced anxiety symptoms
Pruessner et al. [92]	Systematic review	Online therapy platform	Improved eating disorder management
Barac et al. [93]	Cross‐sectional study	Wearable device	Enhanced physiological monitoring
Younis et al. [94]	Meta‐analysis	AI‐driven chatbots	Enhanced counseling support
van't Wout‐Frank et al. [95]	Randomized controlled trial	Virtual reality therapy	Improved PTSD outcomes
Breuer‐Asher et al. [96]	Cohort study	Mobile apps	Reduced stress levels

According to Ogugua et al. [97], digital tools and technology improve mental wellness. These tools include smartphone applications, online therapy platforms, wearable gear, AI technologies, and VR technology. Each type of digital mental health therapy has pros and cons, making the field large and ever‐changing, although no digital resource for mental health promotion is more popular than smartphone apps. They offer mindfulness techniques, mood monitoring, CBT, and psychoeducation. Headspace and Calm provide guided meditations and relaxation exercises to reduce stress, anxiety, and depression [98]. Online platforms like BetterHelp and Talkspace connect people with licensed therapists remotely, allowing them to conduct therapy sessions via text and audio and video materials. This capability makes mental health services more accessible, especially to people in rural and underserved areas. Fitbits and Apple Watches track the wearer's heart rate, sleep patterns, and activity levels to show their general health. These wearables can spot mental health issues early by noticing changes from the norm, allowing for prompt interventions [99]. Woebot and Wysa are AI‐powered chatbots that provide real‐time mental healthcare by reacting to user input and offering customized suggestions using ML and NLP [100]. Therapists may use VR to create immersive experiences to help patients with anxiety, PTSD, and phobias overcome their fears. VR therapies have shown promise in improving traditional therapy by providing controlled and adjustable surroundings. Table 3 shows the results of our thematic analysis, serving as a reference.

**Table 3:** Results of thematic analysis of studies on digital tools for mental health promotion.

Theme	Description
Mobile applications	Common functionalities include mindfulness exercises, CBT techniques, mood tracking
Online therapy platforms	Facilitate remote access to therapy sessions via text, audio, and video
Wearable devices	Monitor physiological metrics like activity levels, sleep patterns
AI‐driven tools	Provide personalized support through natural language processing, machine learning
Virtual reality (VR) technologies	Used in exposure therapy, creating immersive environments for anxiety and trauma‐related disorders
Integration with traditional therapy	Complementary role of digital tools in enhancing accessibility and effectiveness of in‐person therapy
Personalization and AI	Adapt digital tools to individual user needs, improving engagement
Holistic health monitoring	Offer comprehensive insights into physical, emotional, and behavioral aspects of mental health
High user engagement	Positive feedback on convenience, accessibility, and immediate support
Positive mental health outcomes	Reported reductions in stress, anxiety, depression symptoms
Challenges	Data privacy concerns, disparities in access, need for long‐term efficacy evidence

This study provides a thorough, evidence‐based assessment of digital mental health technologies' usefulness, limitations, and trends. We systematically examined how smartphone applications, AI‐driven therapies, wearable devices, and VR‐based therapy may improve mental healthcare. The results focus on digital mental health therapy's accessibility, effectiveness, and sustainability.

### 6.1 Effectiveness of Digital Mental Health Interventions

The results show that digital mental health therapies are improving stress, anxiety, and depression in many populations. Studies show that CBT smartphone applications enhance mental health. Users had higher self‐esteem, better stress management, and greater treatment adherence [101, 102]. Wysa and Woebot, as AI chatbots, use NLP and ML to provide real‐time, tailored treatments, increasing accessibility. The findings suggest that these digital therapies enhance psychotherapy, increase engagement, and provide scalable mental health solutions. The results demonstrate VR therapy's potential for revolutionizing exposure‐based social phobia, PTSD, and anxiety disorder treatments. VR‐based treatments replicate controlled circumstances to gradually desensitize patients to anxiety‐inducing stimuli, improving therapeutic outcomes over traditional exposure therapy [103]. VR is not extensively used because it is costly to create, particularly in resource‐poor areas.

### 6.2 Accessibility and Digital Equity Considerations

This study suggests that digital mental health therapies improve mental healthcare in poor communities. AI‐powered mental health solutions and mobile applications have decreased traditional therapy's drawbacks, namely cost, stigma, and location barriers. However, earlier studies show a digital divide, with low‐income and less literate people struggling to use these therapies [104, 105]. This study emphasizes the necessity for laws that ensure digital mental health services are provided to everybody, particularly in developing nations. Mental health technology should be incorporated by public health campaigns, corporate wellness initiatives, and educational institutions to bridge this gap and establish inclusive digital mental health ecosystems.

### 6.3 Sustainability and Ethical Implications of Digital Mental Health Technologies

Digital mental health solutions provide significant advantages, but the results highlight sustainability issues that must be addressed for long‐term success. Data privacy and security are key concerns because digital interventions collect user data. AI‐powered mental health solutions' algorithmic bias raises ethical questions about accuracy, fairness, and inclusiveness [106]. User engagement and retention remain key barriers to digital mental health therapy adoption. Because many AI‐powered tools and mobile apps have high initial engagement but low long‐term adherence, more interactive, tailored, and flexible intervention approaches may be needed to retain users.

Flett et al. [107] used the Headspace app to analyze university students' stress and health. Their randomized controlled investigation included 150 participants using the app for 10 min daily for 8 weeks. The software reduced stress and increased mindfulness and pleasure, suggesting it may be a scalable mental health solution. Marcelle et al. [54] examined BetterHelp's online anxiety and depression treatment platform. Their study enrolled numerous participants in weekly text and video conference therapy sessions for 3 months. A substantial percentage of users were satisfied with the platform's availability and convenience of use, and anxiety and melancholy decreased significantly. Ng et al. [108] studied how Fitbits influenced patients with severe mental disorder over time. By demonstrating that more physical activity and better sleep reduced sadness and anxiety, wearable technology showed its potential in mental health therapy. Jang et al. [109] piloted Woebot chatbot‐based CBT for youths. The participants liked the AI‐powered tool and saw significant anxiety and depression benefits. A meta‐analysis by Stein et al. [110] examined VR‐based anxiety management. VR treatment decreased anxiety symptoms similar to exposure therapy, highlighting VR's potential as a unique therapeutic technique.

Integrating digital technology with traditional therapy is popular. Digital technology may enhance in‐person therapy sessions, making them more accessible and practical. AI and ML have improved the customization of mental health therapy. AI‐powered systems may learn from user needs for engaging, tailored help [111]. Wearable electronics and health monitoring apps that monitor mental, emotional, and behavioral health have increased. Research demonstrates that digital mental health aids, notably AI‐powered chatbots, have an extensive and active user base. They are simple to use, accessible, and offer fast relief; therefore, people praise them [112]. Research shows that digital treatments for mental health improve well‐being, stress, anxiety, and depression. More consumers are choosing digital solutions featuring professional help and self‐guided resources. Users value the privacy and adaptability offered by digital platforms [113].

Despite several studies showing its short‐term benefits, few have examined digital mental health therapy's long‐term efficacy [114, 115]. The effectiveness of digital technologies in marginalized and diverse communities needs more study. Despite concerns, few studies of rules and best practices to safeguard user information in relation to digital mental health tools exist [116, 117]. This study shows that online mental health tools have great potential. These technologies provide innovative, accessible, and cost‐effective mental health therapy for various individuals and circumstances. The technology divide, privacy concerns, and the requirement for convincing evidence must be addressed to benefit from digital mental health therapy. New technology will increase opportunities for digital innovation in mental healthcare. This study emphasizes the importance of employing these technologies to meet mental healthcare needs and improve well‐being.

## 7 Discussion

This study expands the literature on digital mental health interventions by rigorously analyzing their effectiveness, accessibility, and sustainability. The results are presented in the context of the academic literature and compared with those of previous studies. This study supports prior studies suggesting digital mental health therapies may enhance mental health [118, 119]. Mobile CBT applications may reduce depression and anxiety symptoms [120]. This study demonstrates that AI‐powered digital therapy tools and chatbots can replace traditional mental healthcare at low cost. Our research supports past studies' findings that digital treatments improve mental healthcare access, particularly in underprivileged and rural areas [121, 122]. Previous studies have clarified VR‐based therapy's role in anxiety disorder exposure treatment [123, 124]. Our study shows that VR therapies increase treatment adherence and emotional regulation in patients with PTSD. Affordability and technological infrastructure remain barriers to VR adoption. Disparities in digital mental health tool usage and duration are apparent. This study contradicts Schwartz et al. [125], who claimed digital therapies have good user adherence over time. Engagement rates fall dramatically after early uptake. Digital mental health solutions are popular but only work if people use them regularly. We must discover ways to engage individuals. Most studies have focused on Western populations, but this one highlights the global digital divide, cultural acceptability, internet accessibility, and digital competence in technology‐based mental health therapies [126, 127]. If digital mental health products are to be helpful globally, they require culturally sensitive design frameworks and localized adjustments. The literature has overlooked ethical and regulatory issues in digital mental health technologies, but this study addresses them. To sustainably incorporate digital mental health solutions into mainstream healthcare systems, we must address algorithmic bias in AI‐driven treatments, data privacy challenges, and the lack of regulatory frameworks. This study also stresses combining digital and in‐person therapies as hybrid mental health methods. Integrated care approaches may enhance mental health outcomes more than digital technologies, although technology supports traditional therapy rather than replacing it.

Digital tools and technology may promote mental health via accessible, cost‐effective, personalized therapy, helping traditional mental health treatments overcome issues with financing, cost, and societal stigma. This study on digital mental health highlights the need to leverage technology to meet the growing demand for mental health services and improve well‐being. Digital mental health technology may help persons without easy access to mental healthcare. Cultural stigma, geographical isolation, and a shortage of mental health experts make mental health treatment difficult in many countries, including low‐ and middle‐income ones. Digital technologies that provide remote therapy, self‐help, and support groups may offset this disparity. BetterHelp and Talkspace allow consumers to seek mental health treatment online by linking them with licensed therapists. Users may use Calm and Headspace for mindfulness activities and guided meditations anytime and anywhere. In addition, digital mental health resources are cheaper than in‐person therapy. Because of the cost, many individuals cannot afford mental healthcare. Digital technology may reduce costs by providing cheaper alternatives to in‐person therapy. Online subscriptions may be cheaper than per‐session therapy. Digital self‐help tools and apps are inexpensive or unrestricted so that more people can use them. Cost‐effectiveness is essential for mental health treatment to be accessible to all socioeconomic groups. Individualized support is another advantage of digital mental health treatments. AI and ML provide customized goods that learn from consumers' behaviors and preferences.

## 8 Future Directions and Recommendations

The field of digital mental health is poised for significant growth and innovative advancements in the coming years, which will unquestionably transform the accessibility and provision of mental health treatment. Advances in technology will enhance digital mental health treatments' effectiveness, accessibility, and user experience. More research is needed to understand digital mental health treatments' pros and disadvantages to establish their efficacy and long‐term benefits. Various digital therapies work, according to RCTs, longitudinal studies, and meta‐analyses. However, researchers must study the mechanisms of action to understand why and how the tools work and who benefits most from them. Understanding user engagement and retention is crucial to digital mental health services' success. Research on user preferences, motives, and engagement hurdles should improve user experience and adherence. Gamification, personalization, and user‐centered design may also improve tools. Research should focus on personalizing and adapting digital interventions to match individual needs and preferences. ML algorithms and AI may improve digital tools by leveraging user data to tailor interventions. Research must examine how personalized procedures affect outcomes and whether adaptive therapies that adjust to user input and development are feasible. Assessing cost‐effectiveness and scalability is essential for widely adopting digital mental health treatments. Researchers should compare digital therapy costs and outcomes with those of traditional mental health treatment to establish their economic impact. Studies should also consider scaling digital technologies to ensure widespread and sustainable usage without compromising quality or effectiveness. Digital mental health tools using current technologies may increase therapy usability, accessibility, and effectiveness. Digital technologies like AI, VR, and ML may provide individualized, entertaining, and effective mental health therapy. Developers, politicians, and healthcare practitioners must work together to overcome critical difficulties with laws, accessibility, integration, education, and collaboration to maximize these technologies. Digital mental health products must be studied in terms of their usefulness, engagement, personalizability, and cost‐efficiency. This study will influence standards and best practices. Digital technology can enhance mental healthcare and lives globally if we accept innovation and collaborate.

This section evaluates important growth areas and gives recommendations for developers, healthcare practitioners, and policymakers to ensure they incorporate accessible, effective, and safe digital technology into regular mental health therapy. Cutting‐edge technologies like ML, VR, and AI may lead to more interactive and individualized digital mental health assistance. AI might transform digital mental health treatments. It can automate monotonous tasks, enhance mental health assessments, and provide tailored recommendations. Wysa and Woebot, AI‐powered bots, may chat with users, provide CBT, and measure mood changes using NLP. For patients with mental health challenges who cannot afford or obtain traditional care, these chatbots may be lifesaving. AI can also help clinicians find trends and patterns in massive data sets to predict mental health outcomes and customize therapy. VR has been effective in treating phobias, anxiety disorders, and PTSD. Its realistic simulations make it a viable and safe modality for anxiety treatment. VR exposure treatment with a therapist may help individuals overcome the fear of heights or public speaking. VR makes mindfulness and relaxation more engaging and interactive. Machines trained on user data may discover trends and patterns that people overlook. Developers may use these data to provide more personalized digital mental health therapy. ML can identify physiological changes in wearable device data that indicate stress or concern. Early detection and appropriate therapy, like mindfulness or guided breathing, may help patients manage their symptoms using digital tools.

Digital mental health tools must be integrated into everyday mental health therapy by healthcare providers and politicians to attain their full potential. Integration requires addressing several crucial issues. First, digital mental health technologies need explicit norms and standards to ensure security, efficacy, and safety. Lawmakers should establish high‐quality and practical digital technology development, testing, and usage standards. Methods for RCTs and other studies are needed to assess digital therapy efficacy. Data security and privacy must also be included in policies to protect user data. Second, we must ensure equitable access to digital mental health services for everybody, regardless of geography, money, or computer skills. To assist poor communities in narrowing the digital divide, healthcare providers and governments should collaborate. Such collaboration may be achieved by building user‐friendly, multilingual tools, subsidizing internet access and digital devices, and teaching digital literacy. Digital tools must be inclusive and culturally responsive to satisfy diverse interests and needs. Third, digital mental health technology should complement traditional mental healthcare. Medical experts should employ digital technology to improve traditional therapies. Physicians may use digital tools to follow patients' progress between consultations, give additional aid and resources, and improve communication. Integrating digital and traditional approaches to deliver more comprehensive and customized treatment may improve mental health outcomes. Fourth, to successfully use digital mental health technology, the public, healthcare professionals, and patients must understand its pros and cons. Healthcare professionals should be trained on the ethical and appropriate use of digital technology, considering its pros and cons. Patients should learn about digital resources, including how to use them and the importance of mental health. Public awareness campaigns may increase the use of digital technology as a helpful aid and reduce mental health stigma. Finally, digital mental health requires collaboration among many parties including doctors, professors, politicians, and technologists. Collaborations may help create better and more creative instruments by sharing resources, talents, and knowledge. Tech companies and mental health institutions may collaborate to create innovative, clinically backed solutions. Research partnerships may help produce data on the ability of digital initiatives to impact best practices and standards.

## 9 Conclusions

This study assessed the effectiveness, risks, and accessibility of digital mental health treatments such as smartphone applications, AI‐powered chatbots, wearable electronics, and VR. The study suggests that digital technology may improve mental health management by making treatments more accessible, customizable, and real time in nature. However, issues with data privacy, long‐term user engagement, and access inequalities exist. Evidence suggests that specific digital mental health treatments reduce stress, anxiety, and depression. However, more research is needed to evaluate their long‐term efficacy and amenability to integration into healthcare systems. Wysa and Woebot, AI‐powered chatbots, can adjust recommendations and interventions to user mood and speech patterns. Wearable devices like Fitbit may track heartrate and sleep patterns to deliver tailored insights and actions. Personalized digital mental health solutions work better because consumers receive the support they need. Digital mental health technologies have tremendous benefits but also specific challenges. One primary concern is the digital divide. Helping poor populations access resources is necessary so that everyone can benefit from digital mental health treatments. Device accessibility, internet connectivity, and digital literacy teaching are viable solutions. Privacy and security are equally vital. Digital technologies collect and preserve sensitive personal data, raising concerns about data breaches and exploitation. Digital mental health solutions must meet privacy requirements, and developers and policymakers must work together for secure data. Methods to protect user data include encryption, secure data storage, and open data usage restrictions. Digital mental health tools require robust efficacy and safety evidence. Data support these therapies' efficacy, but additional research is needed to assess their long‐term risks and benefits. RCTs, longitudinal research, and meta‐analyses inform best practices and recommendations.

To maximize the benefits of digital mental health therapy, policymakers and industry stakeholders must aggressively address the current issues and enhance their implementation. Governments should create legal frameworks for data protection, AI ethics, and security to ensure the safe deployment of digital mental health therapies. Public health initiatives should promote low‐cost internet and digital literacy to abolish the digital divide, particularly in underprivileged areas. Multidisciplinary collaboration between mental health professionals, AI experts, and lawmakers may provide more effective, user‐friendly, and evidence‐based digital solutions. Integrating digital technologies into healthcare systems may expedite and scale mental health treatment. Policymakers should promote hybrid therapeutic models that blend digital and traditional therapies. Governments and businesses should finance research on digital mental health therapy's usability, safety, and long‐term benefits. Legislation should be passed to ethically optimize digital mental health therapies in response to rising demand. This study contributes to the discussion on digitalizing mental health and paves the way for future research and policy efforts.

New technology will increase digital innovation opportunities in mental healthcare. With VR, AR, and AI, mental health therapy may become more immersive and compelling. VR is used to create realistic exposure therapy simulations to help patients confront their fears. Augmented reality may enhance relaxation and mindfulness by superimposing virtual settings over real ones. AI‐driven systems may improve customization and prediction by monitoring behavior or physiological indications and providing early remedies. ML algorithms that evaluate trends and patterns in vast amounts of data may improve treatment approaches. Digital technology combined with traditional mental health treatment may allow the creation of more comprehensive mental health plans. This study emphasizes the importance of digital mental health tools for enhancing well‐being and satisfying the growing demand for mental health services. Developers, healthcare providers, and policymakers must collaborate to address digital technology's risks and opportunities. Aims include clear regulations and guidelines, collaborations and partnerships, education and training, and integrating digital technology with traditional care, accessibility, and equality. Digital technologies and technology may enhance mental health therapy and make it more effective and accessible. Moving ahead, we must design, investigate, and deploy digital solutions that address the diverse needs of individuals and communities to make mental health treatment accessible and effective for everyone.

Future studies should monitor individuals to assess how digital interventions improve mental health. Hybrid approaches that combine digital and traditional mental health therapies are needed to provide more comprehensive care systems. Comparing the efficiency of digital therapies across diverse groups may help researchers adapt these technologies to different demographic and cultural situations. Data confidentiality, algorithmic bias, and user confidence in automated therapies are the top AI‐related mental health ethics concerns needing further study. Digital mental health interventions are gaining support, although the research included in this review has limitations. Long‐term follow‐up data are usually unavailable, making it hard to evaluate these therapies. Many studies report self‐reported outcomes, which makes evaluating effectiveness difficult and introduces response biases. Research methodologies, sample sizes, and technological implementations can also differ, making direct comparisons difficult. Technology illiteracy and poor internet connections remain key barriers to accessibility. Ethics in relation to algorithmic biases, user confidentiality, data privacy, and AI‐driven tool use need further investigation. Future research must address these limitation to increase the evidence base and mainstreaming of digital mental health therapies.

## Author Contributions

**Muhammad Khalid Anser:** conceptualization (equal), data curation (equal), formal analysis (equal), funding acquisition (equal), investigation (equal), project administration (equal), resources (equal), validation (equal), visualization (equal), writing – original draft (equal), writing – review and editing (equal). **Agha Amad Nabi:** conceptualization (equal), data curation (equal), formal analysis (equal), funding acquisition (equal), investigation (equal), project administration (equal), resources (equal), validation (equal), visualization (equal), writing – original draft (equal), writing – review and editing (equal). **Ishfaq Ahmad:** conceptualization (equal), data curation (equal), formal analysis (equal), investigation (equal), project administration (equal), resources (equal), validation (equal), visualization (equal), writing – original draft (equal), writing – review and editing (equal). **Muhammad Moinuddin Qazi Abro:** conceptualization (equal), data curation (equal), formal analysis (equal), investigation (equal), project administration (equal), resources (equal), validation (equal), visualization (equal), writing – original draft (equal), writing – review and editing (equal). **Khalid Zaman:** conceptualization (equal), data curation (equal), formal analysis (equal), investigation (equal), methodology (equal), software (equal), validation (equal), visualization (equal), writing – original draft (equal), writing – review and editing (equal).

## Ethics Statement

The authors have nothing to report.

## Consent

The authors have nothing to report.

## Conflicts of Interest

The authors have nothing to report.

## Data Availability

Data sharing is not applicable to this article as not data sets are not generated and analyzed during the current study.
